# Comparative Effectiveness of Pediatric Laparoscopic and Laparoscopically Assisted Inguinal Hernia Repairs: A Systematic Review and Meta-Analysis of Randomized Controlled Trials

**DOI:** 10.7759/cureus.100406

**Published:** 2025-12-30

**Authors:** Medhat Taha, Zahra Belghiath Alsayed, Alhanouf Abdullah Almuhalbidi, Shaima Saad Alamri, Lenah Ahmed Alhafezi, Mashael Khalid Hetaimish, Sajidah Ibrahim Alramadhan, Bushra Essa Alabbas, Asma Abdulkarim Boukhari, Mohammed Khattab

**Affiliations:** 1 Al-Qunfudhah College of Medicine and Surgery, Umm Al-Qura University, Al Qunfudhah, SAU; 2 Department of Anatomy, College of Medicine, Mansoura University, Mansoura, EGY; 3 College of Nursing, King Saud bin Abdulaziz University for Health Sciences, Jeddah, SAU; 4 College of Medicine, Qassim University, Buraydah, SAU; 5 College of Medicine and Surgery, University of Jeddah, Jeddah, SAU; 6 College of Medicine and Surgery, King Abdulaziz University, Jeddah, SAU; 7 College of Medicine and Surgery, Imam Abdulrahman Bin Faisal University, Dammam, SAU; 8 College of Medicine and Surgery, King Khalid University, Abha, SAU; 9 Department of Pediatric Surgery, Maternity and Children Hospital, Al-Qassim, SAU

**Keywords:** comparative effectiveness, laparoscopically assisted repair, laparoscopic surgery, pediatric inguinal hernia repair, randomized controlled trials

## Abstract

Among children, surgical procedures most often done include inguinal hernia repair. Although both laparoscopically assisted inguinal hernia repair (LAIHR) and laparoscopic inguinal hernia repair (LIHR) are rather common minimally invasive methods, their relative safety, efficacy, and outcomes are still questionable. This systematic review and meta-analysis sought to examine the efficacy and complication profiles of LIHR and LAIHR in children derived from data from randomized controlled trials. Through March 2025, PubMed, Embase, Scopus, Web of Science, and CENTRAL were searched systematically to find randomized controlled trials comparing LIHR and LAIHR in pediatric patients. Included were studies reporting on hernia recurrence, operative duration, transient hydrocele, wound complications, and recovery outcomes. Random-effects models were used to conduct meta-analyses. Using the American Society of Plastic Surgeons (ASPS) grading scheme for prognostic or risk studies, the degree of evidence and strength of suggestions were evaluated. Sixteen trials with a total of 1,402 pediatric patients were included. Pooled findings showed no statistically significant variation in hernia recurrence rates between LIHR (1.9%; 95% CI: 0-3%) and LAIHR (0.7%; 95% CI: 0-2%) (P = 0.052; I² = 29%). Likewise, no statistically significant differences were found in the rates of transient hydrocele (P = 0.1379) or wound infection (P = 0.4951). Analysis of operative time revealed extreme statistical heterogeneity (I² = 100%), precluding a meaningful pooled comparison. This indicates that operative duration is highly variable and dependent on unmeasured, center-specific factors such as surgical technique and experience, rather than representing a consistent difference between the two broad procedure categories. Both strategies showed outstanding safety profiles and low complication rates. Early recovery results and cosmetic satisfaction were somewhat more positive with LAIHR in some studies. Both LIHR and LAIHR are effective, safe, and cosmetically acceptable ways to repair pediatric inguinal hernia, with no major variances in recurrence or most complication rates. Operative time is not a distinguishing feature between the techniques but is instead highly context-dependent. LAIHR may have benefits in chosen contexts for surgical time and aesthetic results. The method chosen should fit patient's needs, surgical proficiency, and hospital's assets.

## Introduction and background

With an incidence varying from 1% to 5% in full-term infants and as high as 30% in premature newborns, inguinal hernia repair continues among the most often done surgical procedures in the pediatric population [1,2]. Open herniotomy has historically been the standard of care; however, the development of minimally invasive surgery has brought laparoscopic methods promising better vision, less tissue injury, and perhaps quicker recovery [3,4]. Particularly, in bilateral and recurrent situations, laparoscopic inguinal hernia repair (LIHR) and laparoscopically assisted inguinal hernia repair (LAIHR) have become noteworthy as workable alternatives [5,6].

Its capacity to simultaneously examine the contralateral side, shorten operating time in skilled hands, and perhaps lower the chance of metachronous hernias has driven laparoscopy in pediatric surgery [7,8]. Still, there is discussion about the relative advantages of fully laparoscopic procedures versus laparoscopically aided ones, especially in light of surgical time, recurrence rates, complication profiles, and cosmesis [8]. While other studies argue for the thorough benefits of LIHR, especially in experienced centers, some randomized controlled trials (RCTs) have demonstrated positive results for LAIHR in terms of technical simplicity and shorter learning curves [9,10].

Although the evidence is mounting, there is still disagreement about the best minimally invasive technique for pediatric inguinal hernia correction. Heterogeneity in surgical procedures and outcome definitions adds further complexities to the decision-making process when many studies have produced contradictory results. Therefore, a systematic synthesis of the highest degree of evidence randomized controlled studies is warranted to objectively compare the efficacy and safety profiles of LIHR and LAIHR.

Using data from RCTs, this systematic review and meta-analysis seeks to critically assess and contrast the results of pediatric laparoscopic and LAIHRs. The purpose is to give pediatric surgeons a more obvious evidence-based foundation to assist their surgical decision-making.

## Review

Methodology

For the execution and reporting of systematic reviews and meta-analyses, this study used the Preferred Reporting Items for Systematic Reviews and Meta-Analyses (PRISMA) guidelines. To find RCTs comparing pediatric LIHR with LAIHR, a thorough literature search was undertaken. From their beginnings till March 2025, the search covered electronic databases including PubMed, Embase, Scopus, Web of Science, and the Cochrane Central Register of Controlled Trials (CENTRAL). Search words included permutations of "laparoscopically assisted", "pediatric", "children", "inguinal hernia", and "RCT". Boolean operators and MeSH phrases were used to guarantee that the search technique was both sensitive and specific.

Studies were included if they fulfilled the following eligibility criteria: (1) RCT design; (2) pediatric population (patients under 18 years of age) having inguinal hernia repair; (3) direct comparison between LIHR and LAIHR; and (4) reported at least one of the outcomes: recurrence rate, operative time, postoperative complications (including wound infection, hydrocele, or testicular atrophy), or cosmetic satisfaction. Studies concentrating exclusively on open surgery, adult populations, or lacking comparative results were rejected. Two reviewers first assessed titles and abstracts, then thorough full-text reviews were done to establish final eligibility. Any differences were settled either through consensus or by asking a third reviewer.

Using a predesigned data collection form, two reviewers independently performed data extraction. Study characteristics (author, year, nation), sample size, patient demographics, surgical method specifics, follow-up length, and outcome measures were among the extracted data. Study authors were contacted for clarification in cases of missing or questionable data. Using RevMan (version 5.4), meta-analytic data were combined; risk ratios (RR) with 95% confidence intervals (CIs) were calculated for dichotomous outcomes, whereas mean differences (MD) or standardized mean differences (SMD), depending on measurement scales, were used to analyze continuous outcomes. Because of expected clinical heterogeneity, a random-effects model was used. The I statistic was used to evaluate study variance; results >50% indicated considerable heterogeneity. Sensitivity analyses were run by consecutively excluding studies to determine the robustness of the results.

The Cochrane Risk of Bias Tool for randomized experiments (RoB 2) was used to assess domains including randomization, allocation concealment, blinding, completeness of outcome data, and selective reporting in order to determine risk of bias. The American Society of Plastic Surgeons (ASPS) Level of Evidence Rating Scale for prognostic/risk studies further evaluated the quality of the included studies and the general strength of the evidence. Level I evidence included high-quality RCTs with suitable power and consistent findings; Level II and III studies represented moderate and lower-quality research, respectively. Based on the body of evidence, we used the ASPS Grading Recommendations to determine the strength of clinical recommendations, categorizing them as strong (Grade A), moderate (Grade B), or weak (Grade C).

To guarantee accuracy and methodological strictness, all decisions during participant selection, data extraction, and bias assessment were made independently and in duplicate. Consensus addressed any conflicts and guaranteed objectivity in the synthesis and evaluation of data.

Results

**Figure 1:**
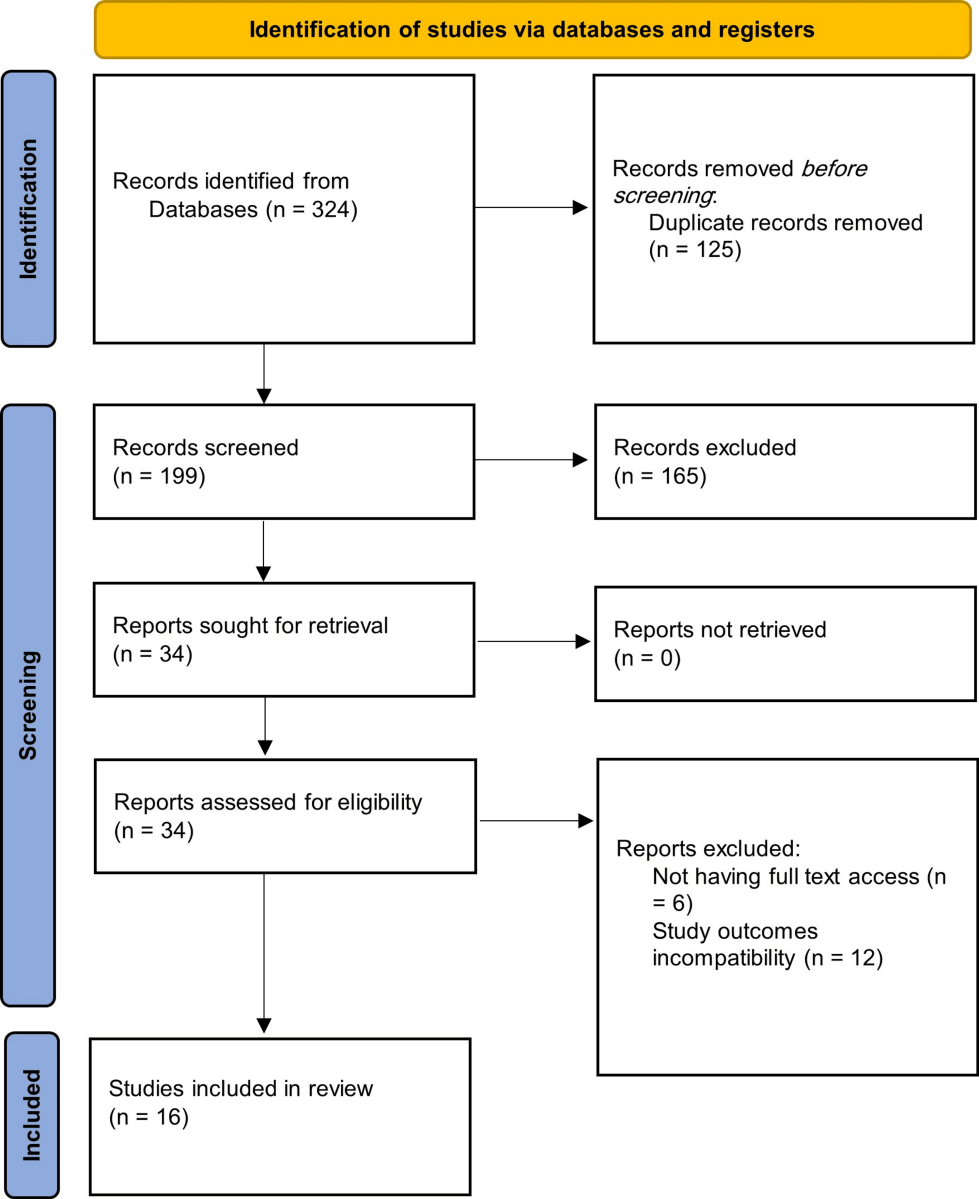
Preferred Reporting Items for Systematic Reviews and Meta-Analyses (PRISMA) flow for including studies

A total of 16 studies were included in the present analysis (Figure 1, [11-26]), comprising RCTs and prospective cohort studies conducted across diverse geographic regions including Egypt [17,22,23,26], India [11,20], Iran [12], Pakistan [16,24], Turkey [13], Japan [25], the USA [18], and others [14,15,19,21] (Table 1). The collective sample encompassed 1,407 pediatric patients undergoing inguinal hernia repair via either laparoscopic (LIHR) or laparoscopically assisted techniques (LAIHR). Age distribution across studies varied widely, ranging from infancy to over five years of age, with mean ages predominantly falling within the first five years of life. The majority of participants were male, with proportions ranging from 58.8% to 100%. Unilateral hernias were more common than bilateral presentations in most studies, though the rates varied substantially, with some cohorts having over 80% unilateral hernias [11,14,17], while others included a significant proportion of bilateral cases [13,23,25]. All studies investigated either LIHR or LAIHR, with the laparoscopic approach slightly more represented in terms of the number of studies and enrolled patients. The design across the studies was largely prospective and randomized, supporting the high internal validity of the included data (Table 1).

**Table 1:** General characteristics of the included studies (author name, country, and study design) and including patients (number, age, gender, and type of hernia) RCT: randomized controlled trial, LIHR: laparoscopic inguinal hernia repair, LAIHR: laparoscopically assisted inguinal hernia repair, NA: not available

Authors	Year of publications	Country	Study design	Intervention	No. of patients	Age (months), mean ± SD	Sex (male), N (%)	Unilateral hernia N (%)	Bilateral hernia N (%)
Mitra A et al [11]	2021	India	Prospective RCT	LIHR	52	NA ± NA, < 12 Y	35 (67.31%)	42 (80.8%)	10 (19.2%)
Fazeli A et al. [12]	2020	Iran	Randomized case-control study	LAIHR	73	28.79 ± 11.45	56 (76.7%)	31 (42.5%)	22 (30.1%)
Celebi S et al [13]	2014	Turkey	Prospective RCT	LIHR	30	98.88 ± 31.2	30 (100%)	0 (0.0 %)	30 (100 %)
Koivusalo A et al [14]	2009	Finland	Prospective RCT	LIHR	47	6 ± NA	36 (76.6%)	47 (100 %)	0 (0.0%)
Igwe A et al. [15]	2019	Nigeria	Prospective RCT	LIHR	34	NA ±NA, 2-156	24 (75%)	29 (85.3%)	3 (8.8%)
Rahman F et al. [16]	2019	Pakistan	RCT	LIHR	50	35.52 ± 30	42 (84%)	35 (70.0%)	5 (10 %)
Abd-Alrazek M et al [17]	2017	Egypt	Prospective RCT	LIHR	132	25.02 ± 8.36	132 (100 %)	106 (80.3%)	26 (19.7%)
Gause C et al. [18]	2016	USA	Prospective RCT	LIHR	26	9.48 ± 11.02	19 (73.1%)	17 (65.4%)	9 (34.6%)
Yao Y et al [19]	2009	Taiwan	prospective cohort study	LIHR	109	64.8 ± NA	75 (68.8%)	52 (47.7%)	38 (34.8%)
Bharathi R et al. [20]	2008	India	Prospective cohort study	LIHR	51	66.96 ±42.24	30 (58.8%)	35 (68.6%)	16 (31.4%)
Chan K et al. [21]	2005	Hong Kong	Prospective single-blinded RCT	LIHR	41	56 ± 45.67	34 (82.9%)	28 (68.3%)	13 (31.7%)
Shalaby R et al. [22]	2012	Egypt	Prospective RCT	LAIHR	125	61.56 ± 28.32	38 (30.4%)	25 (20.0%)	44 (35.2%)
Shalaby R et al. [23]	2023	Egypt	Prospective RCT	LAIHR	230	61.68 ± 33.48	141 (61.3%)	200 (86.9%)	30 (13.1%)
Ahmed A et al. [24]	2022	Pakistan	RCT	LAIHR	148	59.52 ± 37.56	133 (89.9%)	NA	NA
Obata S et al. [25]	2016	Japan	Prospective RCT	LAIHR	109	54.6 ± 32.88	0 (0%)	55 (50.5%)	54 (49.5%)
Shalaby R et al. [26]	2010	Egypt	Prospective RCT	LIHR	75	20.4 ± 13.67	52 (69.3%)	28 (37.3%)	16 (21.3 %)
Shalaby R et al. [26]	2010	Egypt	Prospective RCT	LAIHR	75	23.6 ± 12.99	47 (62.7%)	34 (45.3%)	19 (25.3%)

The pooled analysis across the included studies confirmed favorable safety and efficacy profiles for both laparoscopic (LIHR) and laparoscopically assisted (LAIHR) inguinal hernia repair techniques. Recurrence rates were uniformly low, with study-level data showing rates for LIHR ranging from 0% [13,20] to 3.8% [11] and for LAIHR from 0% [23,25] to 1.6% [22]; pooled analysis found no statistically significant difference (P = 0.052). By contrast, operative duration demonstrated extreme variability, with meta-analysis revealing maximal statistical heterogeneity (I² = 100%). This indicates that operative time is not a consistent property distinguishing the techniques but is instead highly dependent on center-specific factors such as surgical technique and experience. Postoperative complications were infrequent: transient hydrocele was the most common, with an incidence up to 18.2% in one study [17], although typically below 5%, and wound-related complications were rare. Patient-centered outcomes, including cosmetic satisfaction and recovery time, frequently favored minimally invasive approaches, with several trials reporting superior results for LAIHR (Table 2) [12,23,24].

**Table 2:** Outcomes of Interventions including hernia recurrence rate, wound healing, overall results, and complications LIHR: laparoscopic inguinal hernia repair, LAIHR: laparoscopically assisted inguinal hernia repair, NA: not available, SSI: surgical site infection, CPPV: contralateral patencies of processus vaginalis, SILPEC: single-incision laparoscopic percutaneous extraperitoneal closure, LPEC: percutaneous extraperitoneal closure

Authors	Year of publications	Intervention	Hernia recurrence rates N (%)	Surgery duration Mean ± SD	Transient hydrocele rate N (%)	Wound healing N (%)	Other complications N (%)	Duration of hospital stay, Mean ± SD	Recovery time Mean ± SD	Overall positive results (Compared with open surgery)	Level of evidence
Mitra A et al [11]	2021	LIHR	2 (3.85%)	66.98 ± NA	4 (7.69%)	NA	Scrotal edema; 2 (3.85%)	NA ± NA	24 hours	There was significant recovery within 24 hours in the laparoscopic study group (41; 78.85% in laparoscopic vs. 30; 57.69% in open group, p = 0.0204).	Level 1
Fazeli A et al. [12]	2020	LAIHR	2 (2.7%)	22.4 ±5.8	2 (2.7%)	0 (0.0 %)	Bleeding: 2 (2.7%), umbilical hernia at the port site: 5 (6.84%), transient hydrocele: 2 (2.7%), overall postoperative complication rate: 5.4%	33.6 ±8.4	NA ± NA	Shorter operative time compared to open surgery (OS), better cosmetic outcomes with smaller scars, and higher satisfaction level	Level 1
Celebi S et al [13]	2014	LIHR	0 (0.0 %)	32.67 ±3.24	3 (10.0 %)	NA	0 (0.0%)	NA ± NA	57.6 ± NA	Lower pain scores and analgesic requirements and reduced operative and patient recovery times.	Level 2
Koivusalo A et al [14]	2009	LIHR	2 (4.3%)	33 ±NA	NA	NA	37 (79%) required rescue analgesia postoperatively	5 ±NA	57.6 ±33.6	LR required significantly longer operating time and hospital stay.	Level 1
Igwe A et al. [15]	2019	LIHR	0 (0.0 %)	39.3 ±2.31	1 (2.9%)	1 (2.9%)	SSI (1 patient)	NA ±NA	NA ± NA	NA	Level 2
Rahman F et al. [16]	2019	LIHR	2 (4.0 %)	18.3 ±3.1	1 (2.0 %)	NA	0 (0.0%)	40 ±6.8	NA ± NA	NA	Level 2
Abd-Alrazek M et al [17]	2017	LIHR	2 (2.6%)	20.42 ±1.78	24 (18.2%)	NA	NA	Less than 24 h	NA ± NA	Excellent cosmetic results with nearly invisible scars for all patients, minimal postoperative discomfort	Level 1
Gause C et al. [18]	2016	LIHR	NA	27.9 ± NA	NA	1 (3.8%)	Postoperative complication: 3	27.9 ±15	60.96 ± 33.36	Shorter operative time, faster recovery, better cosmetic outcomes, less pain, and low complication rates	Level 1
Yao Y et al [19]	2009	LIHR	1 (0.8%)	70 ±21	0 (0.0 %)	NA	Chronic wound pain impeding exercise (1%)	43.2 ± NA	NA ± NA	The incidence of hernia recurrence was lower.	Level 2
Bharathi R et al. [20]	2008	LIHR	0 (0.0 %)	25.31 ±13.02	2 (5.7%)	NA	2 (5.7%) peritoneal bleed	31 (88.57%) <10 hours, 4 (11.43%) 24 hours	<3 hours: 26 (74.3%), >3 and <6 hours: 9 (25.71%)	Better cosmesis and the ability to detect and simultaneously repair CPPV	Level 2
Chan K et al. [21]	2005	LIHR	0 (0.0 %)	34.0 ± 6.26	1 (2.4%)	NA	Hypertrophic scar: 1, skin sensitivity to dressing: 2	10.66 ± 5.319	48.21 ± 28.68	Less postoperative pain, improved wound healing, faster recovery, and fewer complications	Level 2
Shalaby R et al. [22]	2012	LAIHR	1 (0.8%)	11.4 ± 2.7	NA	NA	NA	5 ± 3.23	6 ± NA	Shorter operative time, faster recovery, fewer complications, better cosmetic outcomes	Level 1
Shalaby R et al. [23]	2023	LAIHR	0 (0.0 %)	37.29 ± 4.68	0 (0.0 %)	NA	In Group A, nine patients (7.8%) experienced postoperative umbilical infections.	< 24 H	NA ± NA	Excellent cosmetic results	Level 1
Ahmed A et al. [24]	2022	LAIHR	1 (0.7%)	24.79 ± 3.44	NA	NA	Two cases (1.4%) had contralateral metachronous hernia.	NA ± NA	NA ± NA	Shorter operative times, a lower incidence of contralateral metachronous inguinal hernia (CMIH)	Level 1
Obata S et al. [25]	2016	LAIHR	0 (0.0 %)	45.16 ± 13.37	NA	7 (6.4%)	Prolonged wound healing: six cases (11.1%), total postoperative complications: nine cases (13.8%)	<24 hours	24 ± NA	SILPEC is feasible and safe, and offers superior cosmetic outcomes, lower postoperative complications.	Level 1
Shalaby R et al. [26]	2010	LIHR	3 (4.0 %)	21.9 ± 7.2	3 (4.0 %)	NA	0 (0.0%)	5 ±3.23	Immediately after surgery	NA	Level 1
Shalaby R et al. [26]	2010	LAIHR	1 (1.3%)	11.4 ±2.7	2 (2.7%)	1 (1.3%)	0 (0.0%)	5 ±3.23	Immediately after surgery	LPEC is known for its simplicity and proven efficacy.	Level 1

Meta-analysis

**Figure 2:**
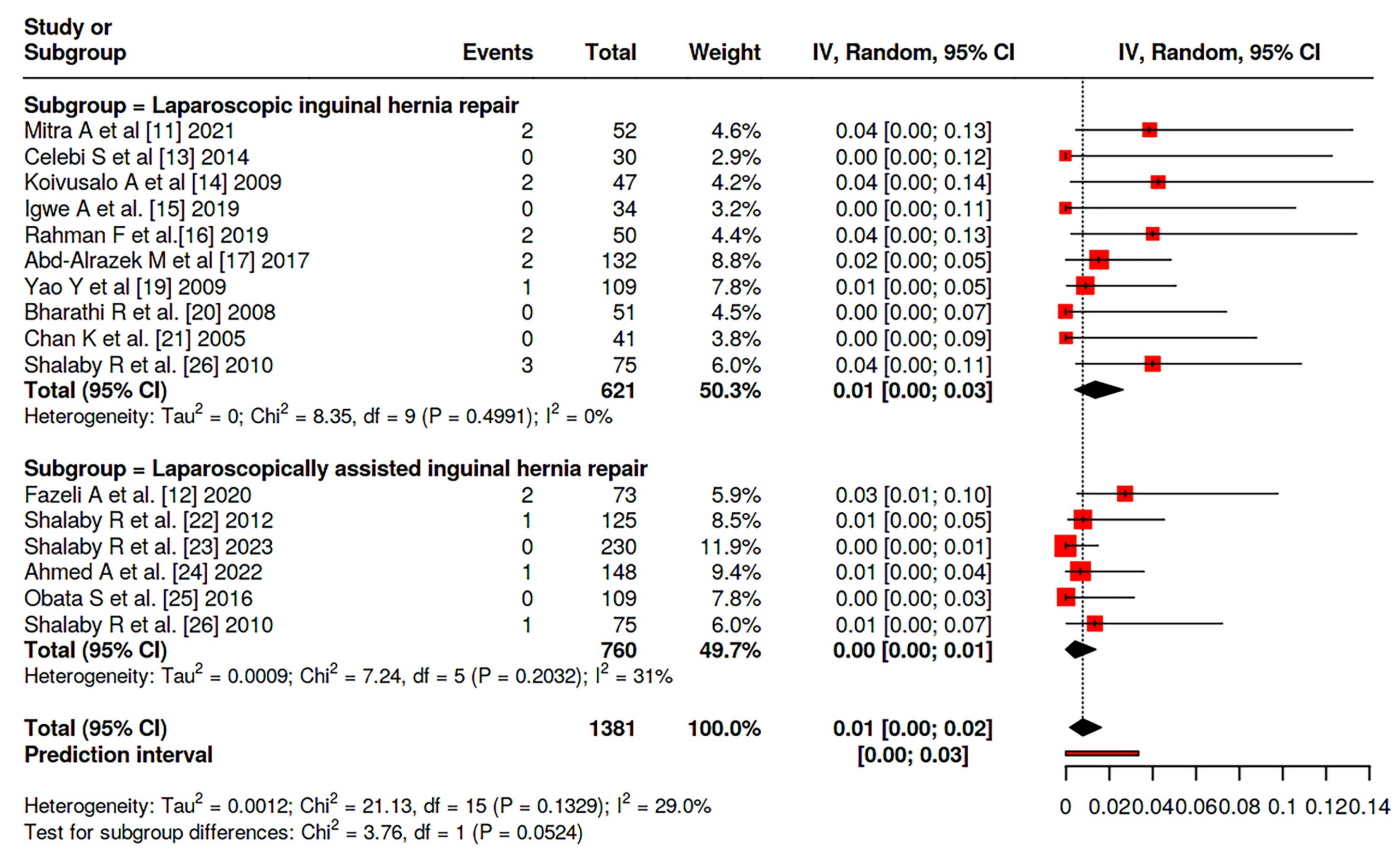
Forest plot of the incidence of hernia recurrence among patients among studies Data pooled from studies [11,12,13,14,15,16,17,19,20,21,22,23,24,25,26]

**Figure 3:**
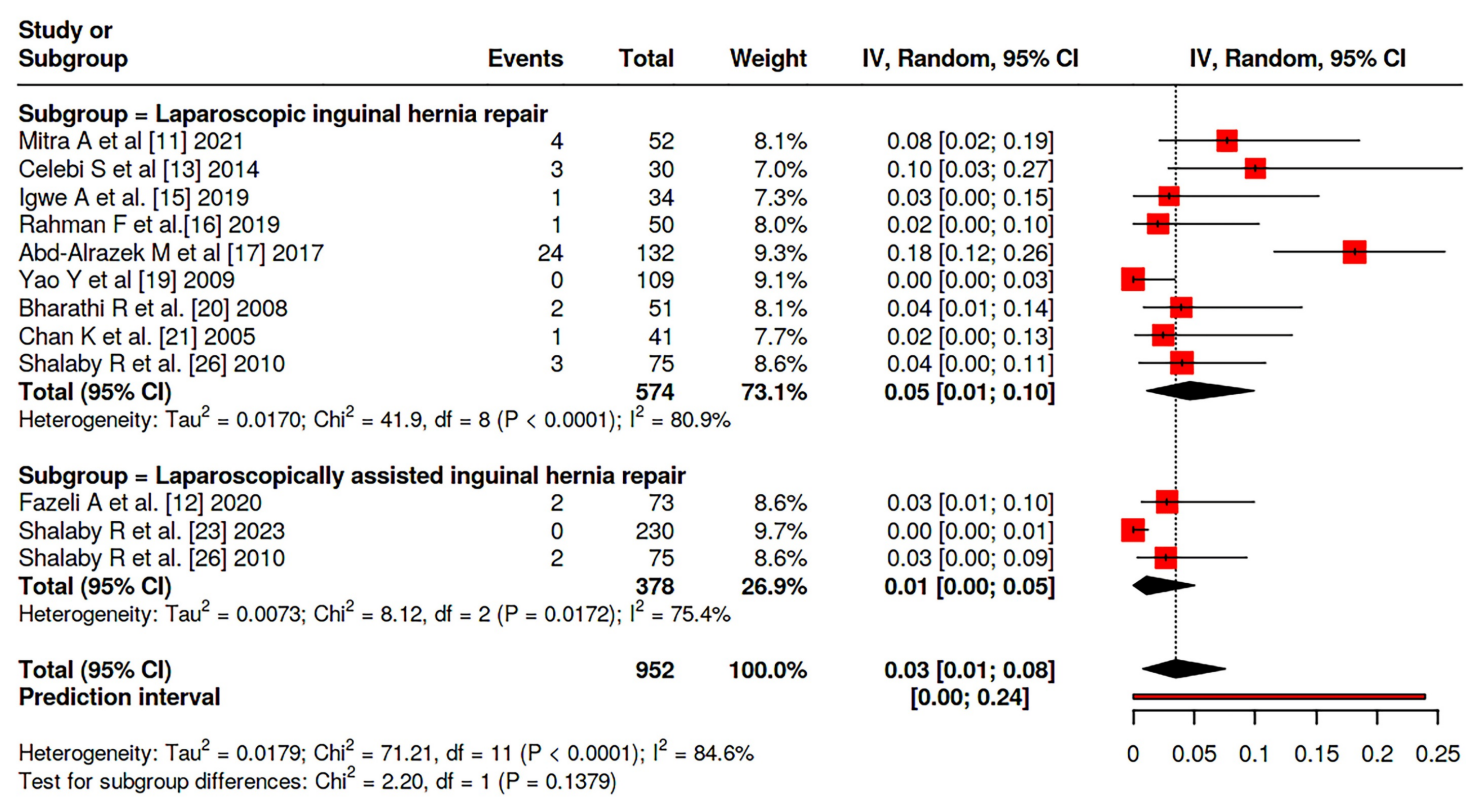
Forest plot of the transient hydrocele rate among the patients among studies Data are pooled from studies [11,12,13,15,16,17,19,20,21,23,26]

**Figure 4:**
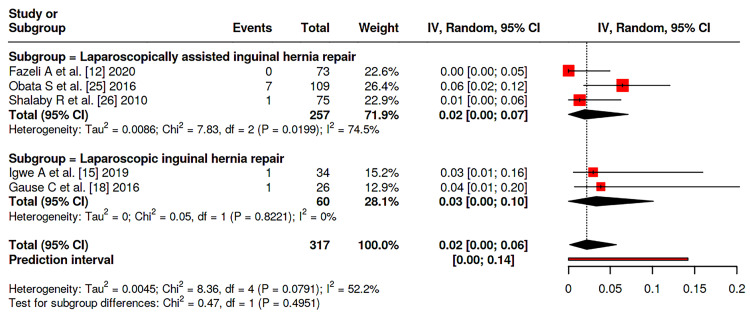
Forest plot of the incidence of wound complications among studies Data pooled from studies [12,15,18,25,26]

**Figure 5:**
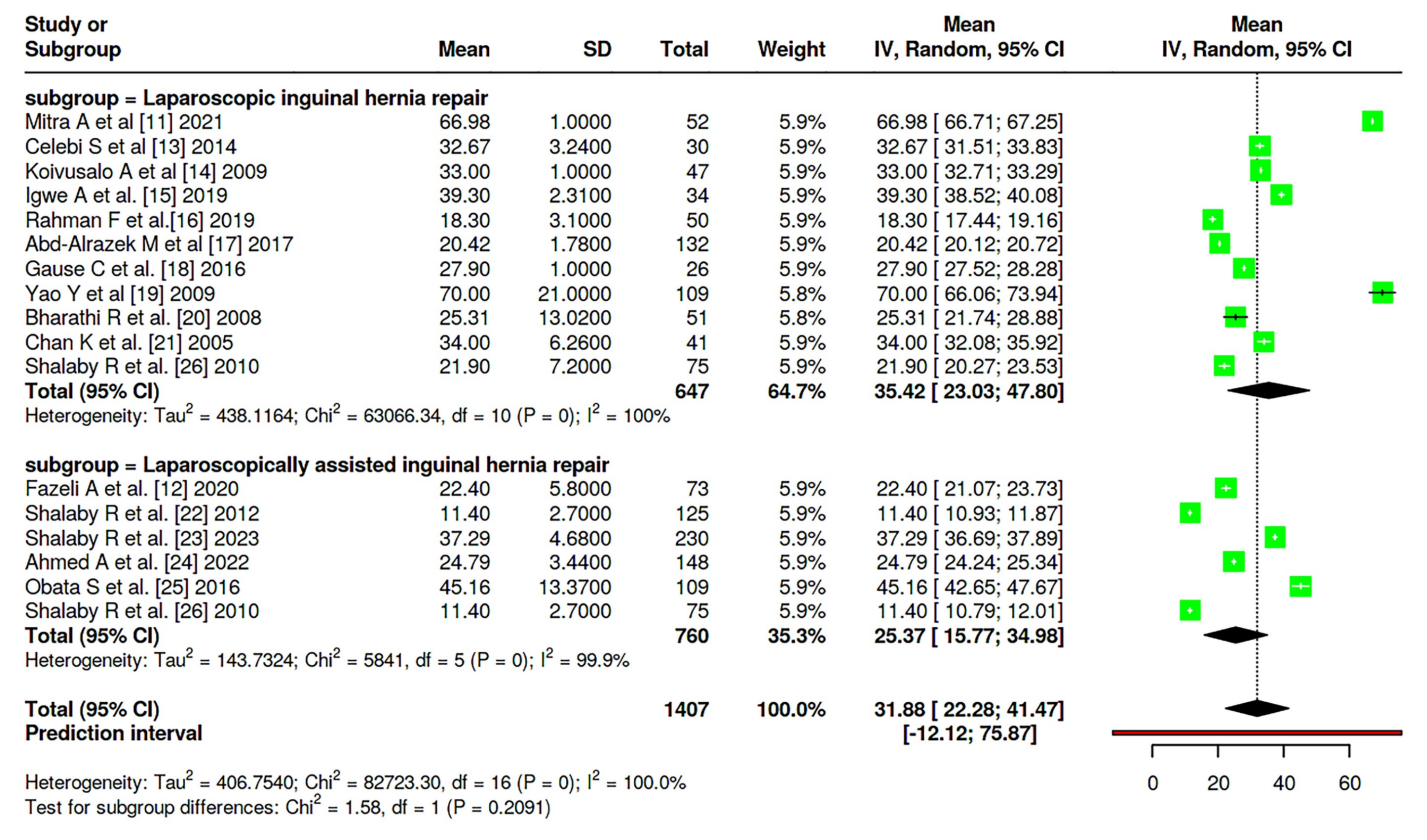
Forest plot of the surgery duration among the patients among studies Data pooled from studies [11-26]

Meta-analysis was conducted among the studies and showed that there is no significant difference between patients on both groups considering the incidence of hernia recurrence (P = 0.052), with rates of 1.9% (95 % CI: 0-3%) in LIHR and 0.7% (95% CI: 0-2%) in LAIHR with nonsignificant heterogeneity between studies (I^2^ = 29%, P = 0.133) (Figure 2). Similary, no significant difference was reported between the two groups considering transient hydrocele rate (P = 0.1379), nor wound infections rates (P = 0.4951), with incidence of 5% (95% CI 1-10%) vs. 1% (95% CI: 0-5%) and 2% (95% CI: 0-7%) vs. 3 (95% CI: 0-6%), respectively (Figure 3,4). Moreover, no significant difference was reported considering operative duration between the two groups (P = 0.2091), with a mean operative duration of 35.42 (95% CI: 23.03-47.80) vs. 25.37 (95% CI: 15.77-41.47) minutes; however, a significant heterogeneity was reported (I^2^ = 100%, P = 0.0) (Figure 5).

Discussion

The results of this systematic review and meta-analysis provide evidence for the safety and clinical efficacy of both LAIHR and LIHR in children. Consistent with previous studies indicating that minimally invasive methods are dependable for pediatric hernia repair when done by expert surgeons, the general recurrence rates were low and similar across both methods [27-29]. In our pooled analysis, the recurrence rate in the LAIHR group (0.7%) was marginally lower than in LIHR (1.9%), but the difference did not reach statistical significance (P = 0.052). This fits prior studies suggesting that both methods provide long-lasting hernia closure with little recurrent risk when executed properly [30,31]. The comparison of operative duration between LIHR and LAIHR warrants nuanced interpretation. While the pooled meta-analysis did not show a statistically significant mean difference (P = 0.2091), the result was characterized by extreme statistical heterogeneity (I² = 100%). This profound variability is the most critical finding regarding surgical time, as it precludes a definitive, generalizable conclusion about one technique being consistently faster. The heterogeneity underscores that operative duration is not an intrinsic property of LIHR versus LAIHR but is instead highly dependent on surgeon-specific factors (experience, learning curve, and technical preference), institutional protocols, and case-mix variables (such as the proportion of bilateral or recurrent hernias). The observation from individual studies that LAIHR may be quicker in certain settings [12,22] likely reflects these contextual factors-including potentially less technological complexity and quicker port access in specific implementations-rather than a universal advantage [32].

Regarding hernia recurrence, our analysis found a low and comparable risk for both techniques. Although the pooled recurrence rate was numerically lower for LAIHR (0.7%) than for LIHR (1.9%), this difference did not reach statistical significance (P = 0.052) and was associated with low heterogeneity (I² = 29%). While this represents a non-significant trend rather than a definitive difference, it underscores the overall safety profile of both methods. Larger, adequately powered randomized trials would be required to determine whether this marginal numerical advantage for LAIHR reflects a true, albeit small, clinical effect or is attributable to chance.

Postoperative complications were infrequent overall. Transient hydrocele, a frequent minor complication, was reported at similar pooled rates between groups (P = 0.1379), though with substantial heterogeneity (I² = 85%). This variability likely reflects differences across studies in diagnostic criteria, the rigor of postoperative surveillance, and the duration of follow-up. Wound-related complications, including infection, were rare and statistically indistinguishable (P = 0.4951). These results reinforce earlier reports that meticulous surgical technique and adherence to sterile principles in minimally invasive surgery yield low postoperative morbidity [33,34].

Regarding patient-centered outcomes, several included studies noted a trend favoring LAIHR in terms of cosmetic satisfaction and early recovery metrics [12,23,24]. This cosmetic advantage, often attributed to smaller or fewer incisions, is a significant consideration in pediatric surgery, where long-term scar appearance and minimal postoperative discomfort are priorities for patients and parents alike [8].

## Conclusions

Our systematic review and meta-analysis, which adhered to current best practices, including the exclusive inclusion of RCTs, rigorous assessment with the Cochrane RoB 2 tool, and appropriate use of a random-effects model, confirms that both LIHR and LAIHR are effective and safe options for the management of pediatric inguinal hernia. The high-quality evidence synthesized demonstrates comparable rates of recurrence, transient hydrocele, and wound complications between the techniques. While a non-significant trend suggested a potential marginal advantage for LAIHR in recurrence, and certain studies favored it for cosmetic outcomes, no consistent, statistically significant superiority was found for either technique across core clinical outcomes. The choice of technique should therefore be guided by surgeon experience, technical familiarity, and specific patient factors-such as hernia characteristics and cosmetic considerations-rather than by an expectation of superior efficacy from one method. Critically, operative time was found to be highly variable and context-dependent, indicating it should not be a primary deciding factor. These findings provide a robust, evidence-based foundation for clinical decision-making. To further refine guidance, future large-scale, multicenter RCTs featuring protocol-driven techniques, consistent outcome definitions, and long-term follow-up are warranted to elucidate subtle differences in recovery profiles, cost-effectiveness, and learning curves.
